# Exploring biogas potential data of cattle manure and olive cake to gain insight into farm and commercial scale production

**DOI:** 10.1016/j.dib.2020.106045

**Published:** 2020-07-19

**Authors:** Shiplu Sarker

**Affiliations:** Department of Manufacturing and Civil Engineering, Norwegian University of Science and Technology, 2815 Gjøvik, Norway

**Keywords:** Biomass, Anaerobic digestion, Biogas potential data, Cattle manure, Olive Cake

## Abstract

This article presents raw data of volumetric biogas and its methane composition obtained from anaerobic digestion experiments conducted under lab scale condition. A commercial biogas industry in Trondheim (Norway) developed interest in using olive cake from a Danish farm (Combineering A/S, Birkerød, Denmark) as a substrate for its existing biogas plant. Moreover, local cattle farm owners wanted to evaluate the possibility of investing on a biogas plant using cattle manure generated on their own farmlands. Accordingly, an evaluation of biogas production potential of these substrates was performed and the obtained data in brief are presented.

Specifications table

**Table:** 

**Subject**	Bioenergy
**Specific subject area**	Biomass for anaerobic digestion.
**Type of data**	Excel spread sheets, tables and images.
**How data were acquired**	An electronic balance (Entris 4202–1S, Sartorius, Epsom, UK) for weighing the samples, an in-situ water displacement apparatus for biogas quantitative analysis, a gas chromatograph (SRI instruments, Torrance, USA) for biogas compositional analysis, an oven for drying biomass to determine total solids, a muffle furnace (Nabertherm, Lilienthal, Germany) for biomass combustion in order to evaluate volatile solids, pH litmus papers for measuring pH,and Microsoft excel in a desktop computer for data record and analysis.
**Data format**	Raw and analyzed.
**Parameters for data collection**	Biogas volume and composition, total solids, volatile solids and pH.
**Description of data collection**	The total and volatile solid contents were calculated by using relevant equations in excel spreadsheet based on the sample data at the following processing steps: weighing, drying, weighing, combusting, and weighing again. The produced biogas in the reactor headspace was collected on a water displacement column resulting in reducing the water height equalled to the amount of measured biogas. The manually recorded biogas volume data were transferred to Microsoft excel spread sheets for analysis and to represent in terms of various other units, i.e., specific yield and daily yield. Furthermore, the data for methane and carbon dioxide contents in biogas were obtained from gas chromatography analysis and treated afterwards in excel spreadsheets.
**Data source location**	**Inoculum** City: Trondheim (63.75° N, 11.92° E), Region: North Europe/Scandinavia Country: Norway **Cattle manure** City: Trondheim (63.67° N, 9.49° E), Region: North Europe/Scandinavia Country: Norway **Olive cake** City: Birkerød (55.83° N, 12.41° E), Region: North Europe/Scandinavia Country: Denmark
**Data accessibility**	Repository name: Insight on biogas potential data of cattle manure and olive cake for stimulating investigation on farm and commercial scale production. Data identification number: Mendeley dataset, Mendeley Data, V1, doi: 10.17632/s9xttsg25b.1 Direct URL to data: https://data.mendeley.com/datasets/kd3y4d4kkx/draft?*a* = 0cb67cef-99df-4596–8eef-799cdd1c8a72

## Value of the data

• The presented data are of extreme importance to the local cattle farm owners in performing preliminary assessment to consider for an investment on a biogas plant. Furthermore, the data facilitate in determining the suitability of olive cake as a supplementary material to a commercial anaerobic digestion plant in Norway.
• Both local community, farm owners, researchers and business stakeholders will be benefitted by accessing the data, as the demonstrated data will allow to make a quick assessment whether or not anaerobic digestion is a convenient option for treating investigated feedstocks to generate renewable biogas.
• The current data give insights into biogas production potential of locally available cattle manure and olive cake. In order to enable more comprehensive assessment relevant to commercialization of a biogas plant, these data will lay a strong foundation for the calculation of many basic parameters of R&D interest using which more complex analyses such as economic and advanced experimental activities dealing with multiple and sophisticated parameter measurements can be developed.
• The presented data can be easily interpreted, exchanged and extracted to strip out basic biogas parameters, which allow comparison with similar data generated through a similar or different methodologies in variable contexts, and thus making them as a valuable R&D reference.

## 1 Data description

**Fig. 1:**
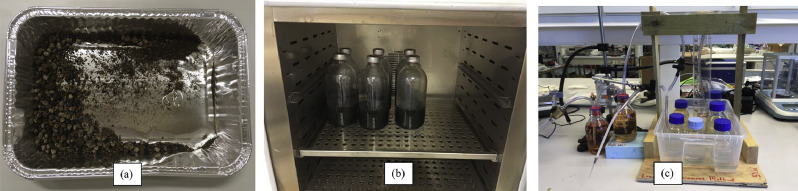
Snapshots of some experimental steps: (a) oven-dried olive cake; (b) incubator with a few reactor bottles; and (c) water displacement apparatus for volumetric biogas measurement.

The data presented in this paper include biogas potential, methane composition, total solids (dry matter) and volatile solids analyses of cattle manure (CM) and olive cake (Fig. 1a.), which were obtained from a local cattle farm in Norway (Ørland, Trondheim) and a Danish industry (Combineering A/S, Birkerød, Denmark) respectively.

Table 1 outlines the description of the materials and important parameters associated with the data.

**Table 1:** Terminologies used in data analysis.

Terms	Descriptions
Inoculum	The organic material containing bacteria used for setting up anaerobic digestion environment.
Substrates	The organic materials used as raw-materials for anaerobic digestion.
Total solids	The total solid component of the substrates and inoculum left after drying.
Volatile solids	The total organic component of the dried substrates and inoculum lost after combustion.
Ash	The inorganic component of the total solids left after combustion.
S:I ratio	The weight ratio between substrate and inoculum.
Anaerobic digestion	The biological degradation of organic substrates at temperature regime suitable to anaerobic microbes' metabolic reactions.
Biogas	The gas produced after series of complex biochemical reactions during anaerobic degradation of substrates.
Cumulative biogas yield	The accumulated total of daily volumetric biogas yield.
Specific biogas yield	The daily volumetric biogas yield per unit mass of total solids or volatile solids.

Table 2 shows the input parameters design considered to set-up experiment. For each substrate to inoculum ratio (S:I), duplicate experiment was conducted and the resulting average data (data for statistical variation are given in supplementary file) are displayed in Table 2.

**Table 2:** Designed input parameters during experimental set-up.

Experimental bottle ID	Material type	Material amount, g			S:I
		Inoculum	CM	Olive cake	
A	Inoculum	200.2	0	0	0
B	Inoculum	200.4	0	0	0
1A	Inoculum + Olive cake	200.4	0	2.1	0.32
1B	Inoculum + Olive cake	199.7	0	2.1	0.32
2A	Inoculum + Olive cake	200.1	0	4.3	0.65
2B	Inoculum + Olive cake	200.2	0	4.2	0.64
3A1	Inoculum + CM	200.1	4.48	0	0.25
3A2	Inoculum + CM	200.0	4.45	0	0.25
3B1	Inoculum + CM	200.3	4.41	0	0.25
3B2	Inoculum + CM	200.1	4.42	0	0.25
4A1	Inoculum + CM	200.2	8.95	0	0.50
4A2	Inoculum + CM	200.3	8.95	0	0.50
4B1	Inoculum + CM	199.8	8.95	0	0.50
4B2	Inoculum + CM	200.1	8.97	0	0.50

Table 3 shows the data for the total solids and volatile solids of inoculum, cattle manure and olive cake. The measurement of total solids and volatile solids were conducted in duplicate, and the resulted statistical variation together with the mean values are given. The standard [1,2] followed for these measurements are also reported (Table 3).

**Table 3:** Data for total solids (TS) and volatile solids (VS).

	Materials			Standard
	Inoculum	CM	Olive cake	
**Total solids,%**	2.97 ± 0.20	6.00 ± 0.50	90.10 ± 1.00	APHA 2005 [1]
**Volatile solids,%**	1.49 ± 0.15	5.67 ± 0.30	85.14 ± 0.20	APHA 2005 [1]

Tables 4 and 5 illustrate the evolution of biogas yield (mL) for CM and olive cake respectively. In these tables, the demonstrated values corresponding to the retention days are expressed with respect to various parameters, i.e., cumulative yield (mL), daily yield (mL/d), specific yield (mL/gTS) and S:I, which are defined in Table 1.

**Table 4:** Biogas potential experimental data for cattle manure.

Retention days	*S:I* *=* *0.25*			*S:I* *=* *0.51*		
	Cumulative yield, mL	Daily yield, mL/d	Specific yield, mL/gVS	Cumulative yield, mL	Daily yield, mL/d	Specific yield, mL/gVS
1	254	254	96	133	133	72
2	453	226	185	200	100	114
3	648	216	274	249	83	145
4	802	200	351	283	71	166
6	1016	169	454	350	58	217
9	1216	135	545	413	46	278
10	1337	134	604	444	44	308
12	1574	131	725	513	43	374
14	1774	127	826	582	42	442
16	1919	120	909	652	41	512
19	2146	113	1041	779	41	615
22	2263	103	1113	852	39	671
24	2263	96	1142	899	37	700
28	2314	85	1175	978	35	736
31	2382	78	1184	1082	35	777
36	2404	68	1195	1317	37	869
43	2431	57	1199	1637	38	986
49	2454	50	1200	1877	38	1072
57	2474	44	1200	2086	37	1148
65	2484	38	1198	2101	32	1153

**Table 5:** Biogas potential experimental data for olive cake.

Retention days	*S:I* *=* *0.32*			*S:I* *=* *0.64*		
	Cumulative yield, mL	Daily yield, mL/d	Specific yield, mL/gTS	Cumulative yield, mL	Daily yield, mL/d	Specific yield, mL/gTS
0	0	0	0	0	0	0
1	145	145	81	178	178	49
2	238	119	133	325	163	90
4	315	79	176	498	124	137
6	368	61	206	593	99	164
7	398	57	222	663	95	183
10	433	43	242	773	77	213
11	458	42	256	808	73	223
14	493	35	275	863	62	238
17	518	30	289	898	53	248
20	543	27	303	953	48	263
22	553	25	309	983	45	271
23	563	24	315	1003	44	277
26	578	22	323	1013	39	280
27	588	22	329	1028	38	284
29	595	21	333	1038	36	287
31	608	20	340	1067	34	295
33	618	19	345	1082	33	299
35	623	18	348	1102	31	304
37	623	17	348	1110	30	307
39	628	16	351	1122	29	310
41	638	16	357	1132	28	313
43	648	15	362	1147	27	317
47	656	14	367	1162	25	321
49	666	14	372	1176	24	325
52	675	13	377	1187	23	328
54	679	13	380	1191	22	329
56	686	12	383	1200	21	332
59	697	12	390	1212	21	335
61	703	12	393	1221	20	337
63	705	11	394	1226	19	339

Besides the biogas potential values, the methane and carbon dioxide content (in%) in biogas throughout the experiment were sporadically measured [3] on a weekly basis and the collected data are displayed in Table 6. Biogas was assumed to compose of CH_4_ and CO_2_ and as accordingly the measured data were normalized, which furthermore organized based on operating S:I ratios.

**Table 6:** Methane and carbon di-oxide content in biogas composition.

Sampling week	Cattle manure				Olive cake			
	*S:I* *=* *0.25*		*S:I* *=* *0.51*		*S:I* *=* *0.32*		*S:I* *=* *0.64*	
	CH_4_	CO_2_	CH_4_	CO_2_	CH_4_	CO_2_	CH_4_	CO_2_
Week 1	58.1	42.0	58.6	41.5	60.3	39.7	59.8	40.2
Week 2	61.7	38.4	60.4	39.6	60.8	39.2	61.1	38.9
Week 3	63.4	36.7	62.2	37.9	62.5	37.5	63.2	36.8
Week 4	66.2	33.9	63.3	36.8	63.1	36.9	64.8	35.2
Week 5	68.5	31.5	66.1	33.9	65.5	34.5	65.2	34.8
Week 6	68.2	31.8	67.4	32.6	69.2	30.8	66.5	33.5
Week 7	67.7	32.4	68.3	31.8	68.2	31.8	68.1	31.9
Week 8	65.7	34.3	65.9	34.2	63.4	36.6	66.2	33.8
Week 9	65.6	34.5	63.5	36.6	61.4	38.6	63.5	36.5

## 2 Experimental design, materials, and methods

### 2.1 Experimental set-up

Anaerobic digestion experiment was set-up as according to the standard ISO 11,734 [4]. Infusion glass bottles of 500 mL (Apodan A/S, Hørsholm, Denmark) were used as anaerobic reactors (Fig. 1b), and during start-up they each were inoculated by adding approximately 200 g of inoculum and different amounts of substrates according to the proportion depicted in Table 2.  The reactors received anaerobic condition by having flushed with N_2_ and afterwards sealed. The set anaerobic temperature was mesophilic (Table 1) at 39 ± 1 °C, which was constantly maintained by a sealed incubator (Fig. 1b) inside which the reactors were kept throughout. The inoculum was collected from Ecopro biogas plant, Trondheim; the cattle manure from a local farm in Trondheim; and olive cake from a Danish company Combineering A/S. Substrates and inoculum weight measurement, when needed, was carried out to nearest ± 0.01 using a sensitive digital electronic balance (Entris 4202–1S, Sartorius, Epsom, UK).

### 2.2 Analytical methods

Total solids and volatile solids were measured analytically, where sample was first weighed, dried in an oven (B9025, Termax, Hagan, Norway) for 24 h at 105 °C, and subsequently combusted in a muffle furnace (LT 5/12, Nabertherm, Lilienthal, Germany) for about 5 h at 550 °C [1,2]. The measured numerical data from each of these steps were then incorporated to relevant equations given elsewhere in Ref. [5] for calculating TS and VS.

Another parameter of interest is pH, which for the reactor bottles was measured using pH litmus strips (Arcol AS, Lørenskog, Norway) for two to three times during the course of the experiment (no data given).

Additionally, the most important parameter, the quantity of biogas produced was analyzed routinely by employing a water displacement appartus [5] (Fig. 1c.), and the recorded data were calibrated to STP (standard temperature and pressure) prior to inclusion in Tables 4 & 5. For biogas volume measurement, the collected biogas on reactor headspace was extracted using a syringe-needle tube connected to an aluminum bag (1 L Tedlar bag, Sigma Aldrich, Darmstadt, Germany) from which the biogas was passed through the inverted glass cylinder, resulting in a volume difference equalling to the amount of biogas channel through the water column. The volumetric biogas yield data were also converted to specific and daily yields and reported along with the cumulative yield in Tables 4 & 5.

Parallel to quantitative analysis, samples were also collected in glass vials (10 mL, Apodan A/S, Hørsholm, Denmark) for qualitative analysis using an *in-situ* gas chromatograph (8610C, SRI instruments, Torrance, USA). The chromatography data in terms of CH_4_ and CO_2_ content are expressed in Table 6. The cumulative biogas yield at the end of the experiment represents the biogas potential of the respective substrate, and the percent methane content illustrates the quality of the obtained potential.

The deep insight into both of these parameters are an essential prerequisite to future research in assessing the possibility of a commercial or farm scale biogas plant design or even to increase the biogas productivity of the existing plants.

## Declaration of Competing Interest

The author declares that he has no known competing financial interests or personal relationships which have, or could be perceived to have, influenced the work reported in this article.

## Appendix Supplementary materials

Image, application 1

Image, application 2
